# Status of Novel Cardiovascular Risk Factor and Cardiovascular Disease Risk in an Urban Cuban Population—A Pilot Study

**DOI:** 10.3329/jhpn.v29i5.8905

**Published:** 2011-10

**Authors:** Arturo Rodriguez-Ojea, Celia Alonso, John W.G. Yarnell, Jayne V. Woodside

**Affiliations:** ^1^Faculty of Medicine, Calixto Garcia, Havana, Cuba; ^2^Hermanos Ameijeiras Hospital, Havana, Cuba; ^3^Centre for Public Health, Queen's University Belfast, Belfast, UK

**Keywords:** Cardiovascular diseases, Carotenoids, Cross-sectional studies, Obesity, Risk factors, Cuba

## Abstract

Cardiovascular disease is the main cause of death in Cuba, yet the prevalence of novel risk factors is not known. To examine the prevalence of risk factors of traditional and novel cardiovascular diseases (CVDs) among an urban Cuban population, a cross-sectional pilot survey was undertaken in Havana city, Cuba. Ninety-seven adults aged 45-60 years registered to receive medical care at a policlinic. The prevalences of rates of CVD risk factors were: hypertension (≥140/90 mmHg) (53.6%), hypercholesterolaemia (total cholesterol >5.2 mmol/L) (47.0%), low high-density lipoprotein (HDL)-cholesterol (<1.03 mmol/L) (64.3%); diabetes (self-reported) (24.6%); metabolic syndrome (ATP III criteria) (58.2%); overweight and obesity (body mass index ≥25 kg/m^2^) (78.0%); current smoking (39.3%); elevated level of C-reactive protein (3 <value <10 mg/L) (32.1%), low lipid-standardized vitamin E levels (<5 μmol/mmol of total cholesterol) (69.6%); and high tHcy levels (>15 μmol/L) (11.1%). The total carotenoid status was independently associa-ted with waist-circumference and risk of diabetes and metabolic syndrome. In this small unrepresentative sample of people aged 40-65 years from Havana city, there was a high prevalence of traditional and novel CVD risk factors. The total serum carotenoid status appeared to be associated with an increased prevalence of CVD risk factors.

## INTRODUCTION

Cardiovascular diseases (CVDs) are the main cause of death in Cuba (1). Although the mortality rates have decreased in the last 15-20 years, the mortali-ty rates are still among the highest in the Americas (2). There is also a high prevalence of overweight and obesity in Cuba (3). The observed reduction in mortality due to CVDs has been less than would have been expected from the reduction in total serum cholesterol levels, and it has been proposed that this could be due to more novel CVD risk factors (4). The proposed novel risk factors for CVDs include the inflammatory marker C-reactive protein (CRP) (5,6) and dietary factors, such as carotenoid and vitamin B status (7,8). However, there is currently no information on the role of these novel cardiovascular risk factors in determining CVD risk in Cuba.

The aim of this pilot study was to assess potentially novel risk factors for CVD in an adult population in Cuba and to determine how these novel risk factors were associated with classical CVD risk factors.

## MATERIALS AND METHODS

### Study design

Ninety-seven adults, aged 45-60 years, receiving medical care in a policlinic (primary healthcare setting) in Havana were recruited. The exclusion criteria were adults with non-atherosclerotic CVD, current pregnancy, women breastfeeding, psychiatric disorders, cardiomyopathy, acute inflammatory conditions, acute malnutrition, systemic rheumatic diseases, gout, known chronic and acute renal failures, nephrotic syndrome, thyroid disorders, cancer, and HIV-positive or AIDS. Eligible participants were invited to participate by three of 15 family doctors employed within the clinic, representing 20% of the total population within relevant age range registered at the clinic. Trained general practitioners and nurses examined them for traditional and novel cardiovascular risk factors. A general questionnaire, including parental and personal medical records, was completed by the family doctor. Physical activity was assessed using the question “Compared to your friends and people of your age, how would you consider your physical activity: less than, same as, more than?” Anthropometric data included height and weight, from which body mass index (BMI) in kg/m^2^ was calculated. Waist-circumference and hip-circumference were measured according to the established protocols, and the waist-to-hip ratio was calculated. Fasting serum samples were collected and stored at −80 °C until analysis. Medical history, including record of psychiatric disorders, gout, acute malnutrition, cancer, rheumatic disease, neurologic and demyelinating disorders, acute and chronic renal failures, nephrotic syndrome, thyroid diseases, and HIV or AIDS, was also noted. Presence or absence of metabolic syndrome was defined according to the Adult Treatment Panel III criteria (9).

### Laboratory methods

Levels of vitamin A and E and carotenoids in serum were assessed using high-performance liquid chromatography (HPLC) with diode array detection following extraction into heptanes (10). CRP was assessed by latex-enhanced immunoturbidimetric assay (Randox Pharmaceuticals, UK) using an ILab 600 biochemical analyzer and using the I-Lab 600 computer software (Instrumentation Laboratories Ltd., Warrington, UK). Serum lipids (total cholesterol, high-density lipoprotein (HDL)-cholesterol, and triglycerides) were analyzed using enzymatic assays (Randox Ltd., Crumlin, NI) on an I-Lab 600 autoanalyser. tHcy was determined by HPLC with detection of fluorescence according to Ubbink *et al*. (11). Serum folate and vitamin B12 were determined using a SimulTRAC-SNB radioassay kit (ICN Pharmaceuticals, California, USA). The QUICKI and HOMA scores, as measures of insulin resistance, were calculated from fasting glucose and insulin measures (12,13). The HOMA score is fasting plasma insulin (mIU/L) x fasting plasma glucose/22.5 while the QUICKI score is equal to 1/[log fasting plasma insulin (μU/mL) + log fasting plasma glucose (mg/dL)].

### Statistical methods

Continuous data were assessed for normality and logarithmically transformed where appropriate. Data were compared between sexes, or according to published cut-off points for increased CVD risk using Student's independent samples *t*-test (continuous variables) or chi-square test (categorical variables). The associations between the continuous variables were examined using Pearson's correlation co-efficients. Multivariate analysis was carried out using general linear modelling (continuous variable outcomes) or logistic regression (categorical variable outcomes). Confounders were included if they were independently associated with the variable of interest, or were known to be associated with the variable from the scientific literature. A p value of <0.05 was considered significant. All statistical analyses were performed using the SPSS software for Windows (version 17.0).

### Ethical aspects

The study was conducted in accordance with the Helsinki Declaration.

## RESULTS

The overall risk factors and the disease-prevalence rates were: hypertension (≥140/90 mmHg) (53.6%); hypercholesterolaemia (total cholesterol >5.2 mmol/L) (47.0%); low-HDL-cholesterol (<1.03 mmol/L) (64.3%); diabetes (self-reported) (24.6%); metabolic syndrome (ATP III criteria) (58.2%); overweight and obesity (BMI ≥25 kg/m^2^) (78.0%); current smoking (39.3%); elevated levels of CRP (3 <value <10 mg/L) (32.1%); low lipid-standardized vitamin E levels (<5 μmol/mmol total cholesterol) (69.6%); and high tHcy levels (>15 μmol/L) (11.1%).

The distribution of demographic variables and traditional CVD risk factors is presented by sex in Table 1 and of more novel risk factors by sex in Table 2. Women had a significantly lower waist-circumference and waist-to-hip ratio and higher HDL-cholesterol but also higher total cholesterol. A higher proportion reported being diabetic and women reported taking less physical activity than men. The folate status was significantly higher in women than in men but the α carotene status was significantly lower in women than in men.

We then explored how closely the novel risk factors assessed were associated with the traditional CVD and diabetes risk factors. Of the novel risk factors assessed, the total carotenoid status was most closely associated with the status of traditional risk factor in unadjusted analyses, being negatively associated with BMI (r=-0.38, p<0.01), waist-circumference (r=-0.36, p=0.01), triglycerides (r=-0.25, p<0.05), and HOMA score (r=-0.26, p<0.05) and positively associated with tHcy (r=0.31, p<0.01) and QUICKI score (r=0.26, p=0.03). The total carotenoid status was significantly lower in diabetics compared to non-diabetics [diabetics 0.592 (1.74); non-diabetics 0.925 (1.68), p=0.01]; lower in those with metabolic syndrome versus those without metabolic syndrome [metabolic syndrome 0.721 (1.69); without metabolic syndrome 0.970 (1.66), p=0.03] and differed among BMI categories, being higher in those of normal weight than in those who were overweight or obese [normal weight 1.221 (1.46); overweight 0.831 (1.63); and obese 0.679 (1.86), p=0.02]. The total carotenoid status did not differ between smokers and non-smokers [non-smokers 0.895 (1.66); smokers 0.740 (1.87), p=0.17].

**Table 1.** Description of demographic variables and traditional CVD risk factors by sex in a middle-aged Cuban population

Variable or risk factor	Males (n _max_ =46)	Females (n _max_ =51)	p value
Age (years)	54.7 (6.0)	54.1 (6.7)	0.61
Systolic blood pressure (mmHg)	139.0 (15.1)	143.6 (23.3)	0.36
Diastolic blood pressure (mmHg)	83.2 (8.4)	85.1 (11.9)	0.48
Hypertensive (>140/90 mmHg)	48.1	57.1	0.47
Not hypertensive (<140/90 mmHg)	51.9	42.9	
Body mass index (kg/m ^2^)	27.5 (4.0)	29.5 (5.9)	0.14
% of normal weight (<25 kg/m ^2^)	26.9	19.0	0.18
of overweight (25-29.9 kg/m ^2^)	50.0	35.7	
% of obese (>30 kg/m ^2^)	23.1	45.2	
Waist-circumference (cm)	98.4 (10.6)	91.7 (12.0)	0.03
Waist-to-hip ratio	0.95 (0.06)	0.85 (0.06)	<0.001
Total cholesterol (mmol/L)	4.96 (1.03)	5.42 (1.03)	0.047
HDL-cholesterol (mmol/L)	0.86 (0.26)	1.03 (0.36)	0.02
Triglycerides (mmol/L)	1.88 (0.66)	1.90 (0.68)	0.89
% of current smokers	32.5	44.9	0.23
% of diabetics	11.1	33.3	0.04
% of metabolic syndrome	55.2	60.5	0.66
Ethnic origin			0.48
White	67.4	56.9	
Mestizo (mixed)	19.6	21.6	
Black	13.0	21.6	
Physical activity			0.02
% perceived self less active than others	34.6	55.0	
% perceived self equally active to others	42.3	12.5	
% perceived self more active than others	23.1	32.5	

Data presented as mean (SD) for continuous variables or % for categorical variables. P value is given for the comparison of males and females. This was determined using an independent sample *t*-test for continuous variables and a chi-square test for categorical variables.

CVD=Cardiovascular disease;

HDL=High-density lipoprotein cholesterol;

SD=Standard deviation

**Table 2.** Description of novel CVD risk factors and biomarkers of nutritional status by sex in a middle-aged Cuban population

Novel biomarker or CVD risk factor	Males (n _max_ =46)	Females (n _max_ =51)	p value
QUICKI	0.35 (0.04)	0.35 (0.05)	0.71
HOMA	2.12 (1.01, 4.66)	1.98 (1.12, 3.56)	0.72
CRP (mg/L)	2.56 (1.34, 4.80)	2.49 (1.12, 5.11)	0.90
tHcy (μmol/L)	10.6 (8.3, 12.4)	9.2 (7.4, 11.2)	0.16
Folate (nmol/L)	19.3 (12.9, 27.5)	24.5 (18.6, 30.6)	0.03
Vitamin B12 (pmol/L)	309.8 (181.0, 471.7)	330.2 (214.0, 471.2)	0.63
Lipid standardized vitamin E (μmol/mmol)	4.53 (1.41)	4.72 (1.63)	0.59
Total carotenoids (μmol/L)	0.85 (0.63, 1.36)	0.85 (0.56, 1.28)	0.99
α carotene (μmol/L)	0.08 (0.05, 0.13)	0.05 (0.03, 0.09)	0.048
β carotene (μmol/L)	0.19 (0.13, 0.31)	0.21 (0.13, 0.34)	0.66

Data presented as mean (SD) for continuous variables, except for HOMA score, CRP, tHcy, folate, vitamin B12, total carotenoid status, α carotene and β carotene status where variables were logarithmically transformed and presented as geometric mean (interquartile range). P value is given for the comparison of males and females. This was determined using an independent sample *t*-test for continuous variables and a chi-square test for categorical variables.

CRP=C-reactive protein;

CVD=Cardiovascular disease;

SD=Standard deviation

**Table 3.** Average increase in CVD and diabetes-related risk factors per one unit increase (on the log scale) in total carotenoid status among men and women in Cuba

Risk factor	Model 1 Mean (95% CI)	p value	Model 2 Mean (95% CI)	p value
BMI	-8.2 (-14.4,-2.1)	p=0.01	-7.9 (-5.9, 0.2)	0.054
Waist-circumference	-21.0 (-34.5,-7.5)	p=0.003	-21.2 (-39.4,-3.0)	0.026
Waist-to-hip ratio	-0.04 (-0.11, 0.03)	p=0.21	-0.05 (-0.17, 0.08)	0.46

Model 1=Adjusted for age, sex, cholesterol, and smoking status;

Model 2=Additionally adjusted for ethnic origin, carotene intake, physical activity, hypercholesterolaemia, and hypertension.

BMI=Body mass index;

CI=Confidence interval;

CVD=Cardiovascular disease

The variables described above in univariate analyses were entered into separate multivariate regression models with the traditional risk factor or condition as the dependent variable and the total carotenoid status as an explanatory variable. These models were initially adjusted for age, sex, cholesterol, and smoking status, and then additionally for ethnic origin, carotene intake, physical activity, hypercholesterolaemia, and hypertension (Table 3). The total carotenoid status was significantly negatively associated with waist-circumference and BMI after initial adjustment, and this remained significant for waist-circumference but just lost significance for BMI (p=0.054) after further adjustment. Similarly, in logistic regression models, with diabetes or metabolic syndrome as outcome variables, the total carotenoid status was significantly associated with risk of being diabetic [Exp(B)=-0.035, p=0.04] or having metabolic syndrome [Exp(B)=-0.035, p=0.035] (logistic regression analyses only adjusted for age, sex, total cholesterol, and smoking status).

## DISCUSSION

This study has examined the traditional and novel CVD risk factor status of urban people aged 40-65 years in Cuba. In general, there was a high prevalence of traditional CVD risk factors in particular, such as hypertension, high cholesterol, low-HDL-cholesterol, and smoking, and a high prevalence of metabolic syndrome. The total serum carotenoid status was the novel CVD risk factor most closely associated with an increased prevalence of CVD risk factors.

Rodriguez *et al*. recently examined the trends in mortality due to coronary heart diseases (CHDs) in the Americas during 1970-2000 (2), and although reduction in mortality due to CHDs was observed for Cuba, the rates of mortality due to CHDs in Cuba were still the highest among women compared to other countries in the Americas and the third highest in men. The authors concluded that recent reductions in mortality due to CHDs were less favourable in Latin America than in the USA and Canada and that this may reflect unfavourable changes in nutrition (including obesity), physical activity, and smoking, together with less-effective control of hypertension and management of CHDs.

Cuba experienced an economic crisis in the early 1990s, triggered by the collapse of the Soviet Union and a reinforced US blockade and characterized by a shortage of fuel, food and essential supplies (4,14). Per-capita energy availability and physical activity increased, and the prevalence of obesity decreased. This was followed by a period of economic recovery, and Cuba now occupies an unusual position as a non-industrialized country with a well-developed public-health sector, which has eliminated epidemic infectious diseases, has low infant mortality, and a long life expectancy (15). To our knowledge, this is the first study to examine both traditional and novel risk factor status in a Cuban population since the nutrition transition in the 1990s.

Even for the traditional CVD risk factors, there are few published data with which to compare our results. Cooper *et al*. have summarized the available data (15). Our overall prevalence rates for hypertension (age-group: 40-65 years; 53.6%) were somewhat higher than that reported in other literature [15-74 years; 25% (16) and (35-64 years; 34%) (17)] but this may reflect the urban centre from which we recruited our sample, the more recent sampling time, and the slightly older age range used. The current smoking status was similar in our population to other reports (15). The obesity rates in the present study were also somewhat higher than what had been reported previously (23% of males and 45% females compared to 8% of males and 14% of females) (16), with reasons for these differences likely to be as for hypertension. Our data relating to lipid concentrations and the prevalence of metabolic syndrome are, to our knowledge, among the first to be reported in the last decade.

For the more novel risk factors, even fewer comparisons with published literature can be drawn. Cut-points for the increased CHD risk remain to be firmly established for many of these factors but for lipid-standardized vitamin E and tHcy, cut-points of 5 μmol/mmol (18) and 15 μmol/L (19) have been suggested respectively. In the present study, using these cut-points, 69.6% and 11.1% of the population can be considered at an elevated risk of CHD based on lipid-standardized vitamin E and tHcy. For CRP, the American Heart Association has suggested that the serum CRP values of >10 mg/L imply the presence of active infection or inflammation but that values between 3 and 10 mg/L are indicative of an increased risk of CVDs (20). Using this cut-point suggests that just under one-third of this population is at an increased risk of CVDs in terms of CRP concentrations.

The consistent associations observed between the total carotenoid status and the traditional risk factors for CVDs and diabetes and risk of diabetes and metabolic syndrome are of interest and have recently been reported in a number of larger epidemiological studies. Akbaraly *et al*. reported that elderly subjects in the highest quartile of total plasma carotenoids had a reduced nine-year risk of dysglycaemia compared to participants in the lowest quartile [relative risk (RR)=0.42, 95% confidence interval (CI) 0.22-0.82], and the same investigators also demonstrated an association with mortality (22). Similarly, in a cross-sectional analysis of middle-aged and older women, Wang *et al*. showed an association between individual plasma carotenoids, although they did not look at total carotenoid status, smoking, obesity, low-density lipoprotein-cholesterol (LDL), HDL-cholesterol, HbA1c, and CRP (23). Finally, Farwell *et al*. demonstrated a cross-sectional association between total plasma carotenoids and current smoking, alcohol ingestion, lipids (total-, HDLand LDL-cholesterol), and ICAM-1 in middle-aged men (24). It is not apparent from any of these studies whether the total plasma or serum carotenoid status is serving as a marker of a diet high in fruit and vegetable consumption, as a marker of other protective lifestyle habits and health behaviours, or whether they are directly having an effect of CVD risk factors but these relatively consistent associations do deserve further exploration.

### Limitations

The present study had several limitations. It was a small and cross-sectional study in nature, and therefore, no causal relationships can be directly assessed. It was also not taken from a truly representative sample of the Cuban population but served as a pilot study of the application of epidemiological research methods and the determination of novel cardiovascular risk factor status in Cuba. For that reason, the conclusions are only valid for the population studied (i.e. patients aged 45-60 years from an urban setting) and cannot be extrapolated to other Cuban populations. As numbers were small, the conclusions must also be interpreted cautiously, as the multivariate adjustment carried out, with adjustment for up to nine covariates, may have produced false significant associations. Nevertheless, it is one of the first studies since the nutrition transition in the 1990s to examine the status of traditional CVD risk factors and the status of novel CVD risk factors. It has also examined how the status of novel cardiovascular risk factors in a Cuban population relates to the status of traditional cardiovascular risk factors.

### Conclusions

In this small unrepresentative population sample, classical CVD risk factors were highly prevalent among people aged 40-65 years in Havana city. The main traditional risk factors present were hypertension, low-HDL-cholesterol, and overweight/obesity while over one-third of this population was at any elevated risk of CVDs through high CRP concentrations and more than two-thirds at an elevated risk in terms of their lipid-standardized vitamin E status. The total serum carotenoid status in this small sample appeared to be inversely associated with an increased prevalence of CVD risk factors.

## ACKNOWLEDGEMENTS

The study was funded by an International Joint Project Scheme award from the Royal Society.
